# Research trends in multimodal metaphor: a bibliometric analysis

**DOI:** 10.3389/fpsyg.2023.1144725

**Published:** 2023-04-17

**Authors:** Zenan Zhong, Suijun Wen, Shukun Chen

**Affiliations:** School of Foreign Languages and Cultures, Guangdong University of Finance, Guangzhou, China

**Keywords:** multimodal metaphor, visual metaphor, pictorial metaphor, bibliometric analysis, web of science (WoS) database, trends

## Abstract

The concept of multimodal metaphor has generated a growing body of literature over the past decades. However, a systemic review of the domain seems to be lacking in relevant literature. This study, therefore, is an attempt to conduct a bibliometric analysis of the field of multimodal metaphor during 1977–2022, with a focus on 397 relevant publications retrieved from the Web of Science Core Collection (WoSCC) with the visualization tool VOSviewer. Some major quantitative findings are: (i) the number of publications in multimodal research began to surge in 2010 upon the seminal work of Forceville’s (2009); (ii) USA, China and Spain are the most productive countries; (iii) journals in the field of advertising, communication and linguistics are important sources of publications; and (iv) eleven clusters of keywords are identified, such as “visual metaphor”, “persuasion”, “pictures”, “impact”, “multimodal metaphor”, “model”, etc., representing crucial areas of interests. We also identified, by qualitative observations, three research trends in multimodal metaphor, driven by cognitive linguistic theory, the theory of pragmatics and visual/multimodal rhetoric theory, respectively. Various theoretical perspectives may shed light on possible further research on multimodal metaphor.

## 1. Introduction

The concept of multimodal/visual/pictorial metaphor has generated a growing body of literature over the past decades since the seminal works of Forceville (1996). Multimodal metaphor, according to Forceville and Urios-Aparisi (2009: 4), is a phenomenon that the target and source are each represented “exclusively or predominately” in different modes, while the visual/pictorial metaphor is considered a phenomenon where the target and source are represented predominately in one mode. However, Eggertsson and Forceville (2009: 430) argued the definition of multimodal metaphor was a “pure” or “strict” metaphor and was “distinguished for analytical purposes only.” They further explained that since “the majority of multimodal metaphors in moving images cue target and/or source in more than one mode simultaneously,” they could be labeled as multimodal metaphors in the broad sense. The definition of multimodal metaphor thus in a broad sense presents its potential to be an umbrella term embracing visual or pictorial metaphors. For the convenience of discussion, we use multimodal metaphor as a general term to name such type of phenomenon in this review.

Multimodal metaphor could appear in various multimodal discourses, such as picture books, posts, magazines, TV shows, films, etc., which comprises different modes (written language, images, sound, gestures, etc.). The interpretation of multimodal metaphors is highly related to traditional metaphor studies. It could be classified into three major dimensions. One is studied within the framework of rhetoric. In rhetoric, the metaphor was used for persuasion or decoration. The representative scholar is Barthes (1977), who first applies the theory to image studies. Another perspective is offered by pragmatics, which considers metaphor as creativity (Sperber and Wilson, 1995) and should be understood with respect to context. Two relevant theories are developed under the perspective: the interaction theory and the blending theory. The interaction theory is developed by Indurkhya (1992). It focuses on “interaction” and tries to invent a relation between the source and one of its activated features. Metaphor interpreted in this approach is often coined as a creative metaphor. The blending theory proposed by Fauconnier and Turner (2002) presupposes that different input spaces merge to create a new “blended space”. It combines selected elements from the input spaces, and as a result yields new, emergent meaning that is not present in either of the input spaces. The third dimension proceeds from a cognitive linguistic perspective. It is inspired by Lakoff and Johnson’s (1980) monograph *Metaphor we live by*. A metaphor is not only a rhetorical device but also a way of thinking and acting, whereas language is just an external manifestation of metaphor (Lakoff and Johnson, 1980). Multimodal metaphor within this theory, according to Forceville (1996), is a strictly directional phenomenon, positing a relationship between pairs of mental representations. It is concerned with entrenched conceptual relationships and how they may be elaborated.

In Forceville’s (2006) review, a number of issues concerning multimodal metaphor research are pointed out. Those issues include, such as the nature of multimodal metaphor, the difference between structural and creative metaphor, how important genre is for the construal and interpretation of metaphor, etc., (Forceville, 2006: 379). To address those issues, more and more scholars have come to extend the multimodal metaphor research to discourses of various genres such as advertising, political cartoons, comics, animation, TV news, films, etc. Those works have not only enriched multimodal metaphor studies but also improved theoretical models due to observations on data of greater varieties.

Despite the significant academic advancement made in multimodal metaphor research by far, the existing pile of literature has received scanty attention of systemic review. An investigation is thus necessary to take stock of the current state of the studies over the past decades. Therefore, this paper conducts a bibliometric analysis, using information visualization methods to make quantitative analysis and observe the indicators of authors, journals, countries, institutions, references and keywords of worldwide literature in a certain field. In this way, we can consolidate the understanding of the nature of multimodal metaphor and propose implications and research directions for future work to promote multimodal metaphor research. Our analysis is guided by the following research questions:

Q1: Who are the most influential authors on the subject of multimodal metaphor?

Q2: What countries/regions and journals are the most influential in the research field of multimodal metaphor?

Q3: What are the most important sub-fields of multimodal metaphor studies?

Q4: What are the research trends and possible future directions in the field of multimodal metaphor?

## 2. Data and methodology

We retrieved the data in our study on Oct 28, 2022 from the Web of Science (WoS) Core Collection Database in all editions excluding Conference Proceedings Citation Index - Science (CPCI-S), Current Chemical Reactions (CCR-EXPANDED), and Index Chemicus (IC). We searched “Topic” with the keywords “multimodal metaphor,” “pictorial metaphor” and “visual metaphor.” The procedure above generated a search result of 397 articles. Then we exported full record and cited references of the 397 studies and imported it to VOSviewer (version 1.6.18) for further analysis. The basic information of all documents including publication year, author, and country is also exported to an EXCEL file for analysis of possible patterns. We have adopted a minimal intervention approach in the data retrieval process to ensure that the quantitative results generated in our research presents least bias.

Following Brika et al. (2022), we have gone through seven steps in the whole study process: study design, research questions, selected types of analysis (co-authorship, co-occurence, citation, bibliographic coupling, and co-citation), data compilation, exportation of basic document information including publication years, author, country/region to EXCEL, visualization (to both network maps in VOSviewer and curve/bar chart in EXCEL) and discussion. We have opted for a relatively low threshold and corresponding weight in visualization in VOSviewer for each type of analysis to present a thorough view of link strength in every network, as shown in Table 1. In the final step, the three authors discussed and illuminated on the quantitative findings for interpretation.

**TABLE 1:** Threshold and visualization for each type of analysis in VOSviewer networks.

Type of analysis	Unit of analysis	Weight in visualization
Citation: country/region	Min number of citations of a country/region = 1	Documents
Citation: source	Min number of citations of a source = 1	Documents
Co-citation: source	Min number of citations of a source = 3	Citations
Co-authorship	Min number of citations of an author = 2	Documents
Citation: author	Min number of citations of an author = 2	Documents
Citation: document	Min number of citations of a document = 1	Citation
Bibliographic coupling: document	Min number of citations of a document = 9	Total link strength
Co-occurrence: key word plus	Mini occurrences of a key word = 2	Occurrence

## 3. Quantitative results

### 3.1. Publication features

#### 3.1.1. Publication years

**FIGURE 1:**
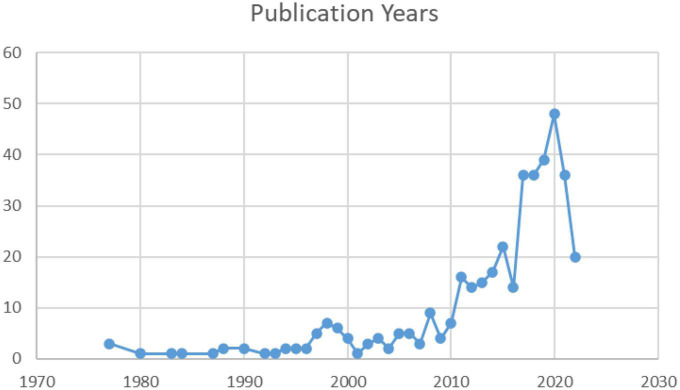
Trends of annual publication of research in multimodal metaphor.

As is shown in Figure 1, the multimodal metaphor research exhibits an overall increasing trend over the decades. Several features are noteworthy here. First, the period from 1977 to 2010 witnessed relatively small amounts of literature in this field with a peak at 7 studies in 1998. Second, the number of papers in the domain of multimodal metaphor began to surge in 2010, and reached a record high at 48 in 2020, since Forceville (2009) brought the term “multimodal metaphor” into the spotlight and began to draw increasing scholarly interests in the field.

#### 3.1.2. Country/region participation

**FIGURE 2:**
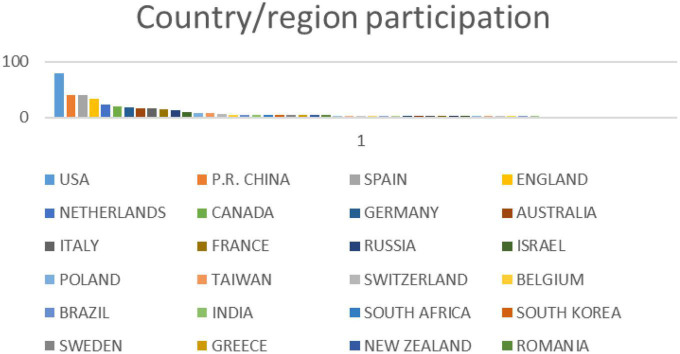
Countries or regions that participated in research related to multimodal metaphor.

A total of 53 countries or regions were involved in research related to multimodal metaphor across the whole world. As shown in Figure 2, there are only 11 countries with more than 10 publications. The most productive countries or regions are USA (80), People’s Republic of China (41), Spain (41), England (34), and Netherlands (23). It is interesting to note that Canada (19), Germany (18), Australia (16), Italy (16), and France (15) share very similar total counts.

**FIGURE 3:**
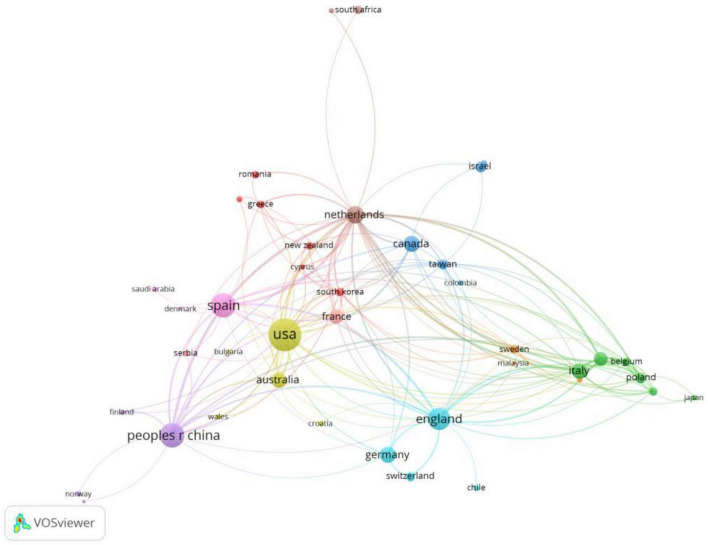
Countries or regions that show collaboration by citation in multimodal metaphor research (min number of citations of a country/region = 1; visualization by weight of documents).

Figure 3 reveals the result from citation analysis in terms of participating countries or regions. The top four countries are USA with a total number of documents of 75, People’s Republic of China with 73, Spain with 41 and England with 34. However, the number of links in England is 20, exceeding that in People’s Republic of China (18) and that in Spain (15). In other words, England is the second most influential country in the number of citation links, while People’s Republic of China and Spain are the third and the fourth. All the countries or regions involved present a certain degree of collaboration with others, albeit with a few of them including Slovenia, Norway, Chile, Lithuania, Saudi Arab and South Africa the least collaborative with only 1 link separately.

#### 3.1.3. Journal participation: citation analysis; co-citation analysis

**FIGURE 4:**
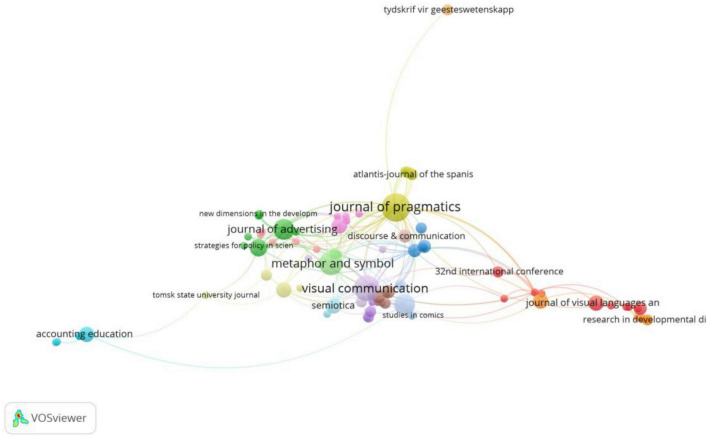
Citation analysis by sources with all sources in account (min number of citations of a source = 1; visualization by weight of documents).

Figure 4 reveals the most important sources of publication in terms of co-citation analysis. According to the assigned total link strength, the most influential journals for multimodal metaphor research are (as shown in Table 2): Année Psychologique (12), Journal of Advertising (44), Metaphor and Symbolic Activity/Metaphor and Symbol (59) (in separate clusters), Journal of Pragmatics (66), Sage Open (7), Accounting Education (9), Monographs of the Society for Research in Child Development/Social Semitoics (6), Multimodal Communication in the 21st century: Professional and Academic (9), Frontiers in Psychology (13), Food Research International (7), Review of Cognitive Linguistics (28), Metaphor and the Social World (13), Visual Communication (44), Semiotica (20), Tydskrif vir Geesteswetenskappe (2) and Discourse and Communication (4). Among all the sources, Journal of Advertising and International Journal of Advertising are clearly the most influential, with a link strength of nine. This is followed by the linkage between Journal of Pragmatics and Visual Communication with a strength of eight. It is worth noticing that the connection between Journal of Pragmatics and Metaphor and Symbol is also strong with a link strength of four. However, when it comes to the number of total citations, Journal of Advertising is the most influential with 358 publications, while Journal of Pragmatics, the second, has 301. The fact that the two journals, Journal of Advertising and Journal of Pragmatics, have such considerable impact shows how the research of multimodal metaphor becomes recognized in the academia of related disciplines.

**TABLE 2:** Journal(s), citation and link strength in the 17 clusters from citation analysis by sources.

Journal	Documents	Citations	Link strength
Journal of Pragmatics	13	301	66
Journal of Advertising	7	358	44
Visual Communication	11	159	44
Metaphor and Symbolic Activity/Metaphor and Symbol	12 (3 + 9)	182 (86 + 96)	59 (27 + 32)
Review of Cognitive Linguistics	7	66	28
Semiotica	4	14	20
Metaphor and the Social World	4	10	13
Frontiers in Psychology	4	25	13
Année Psychologique	1	0	12
Accounting Education	4	28	9
Multimodal Communication in the 21st Century: Professional and Academic	3	18	9
Food Research International	1	7	7
Sage Open	1	0	7
Monographs of the Society for Research in Child Development/Social Semiotics	1/4	28/8	6
Discourse and Communication	3	55	4
Tydskrif vir Geesteswetenskappe	2	0	2

**FIGURE 5:**
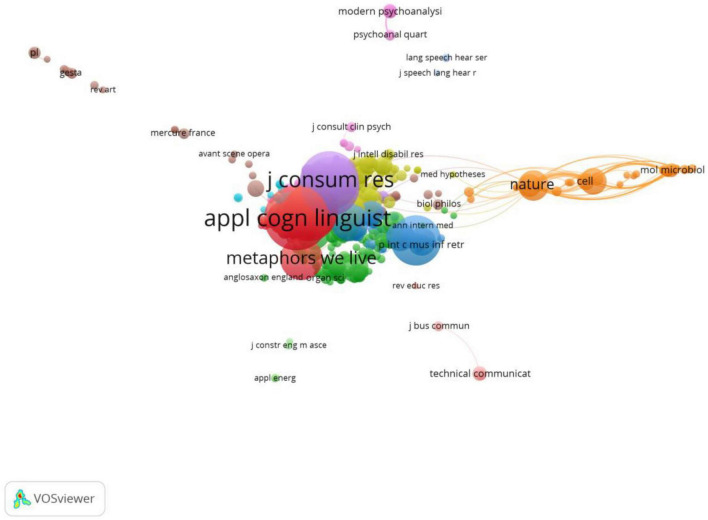
Co-citation Analysis of Cited Sources (min number of citations of a source = 3; visualization by weight of citations).

In addition, it is clear from the network map in Figure 5 that a significant number of these sources are co-cited. As shown in Table 3, five sources seem to hold a dominate position over the others: Applied Cognitive Linguistics (263 citations and 8,665 total link strength), Journal of Advertising (217 citations and 8,600 total link strength), Journal of Consumer Research (215 citations and 7,972 total link strength), Journal of Pragmatics (192 citations and 7,369 total link strength) and Metaphors We Live By (1980) (115 citations and 3,370 total link strength). It can be seen that although Lakoff and Johnson (1980) set out by defining conceptual metaphor and implicated its potential in multimodal meaning making, it is other sources that serve to extend the application of CMT theory in the multimodal fields.

**TABLE 3:** Top five sources according to link strength from co-citation analysis by sources.

Journal	Citation	Link strength
Applied cognitive linguistics	263	8,665
Journal of advertising	217	8,600
Journal of consumer research	215	7,972
Journal of pragmatics	192	7,369
Metaphors we live by	115	3,370

#### 3.1.4. Participating authors: co-authorship of authors, co-citation of authors

**FIGURE 6:**
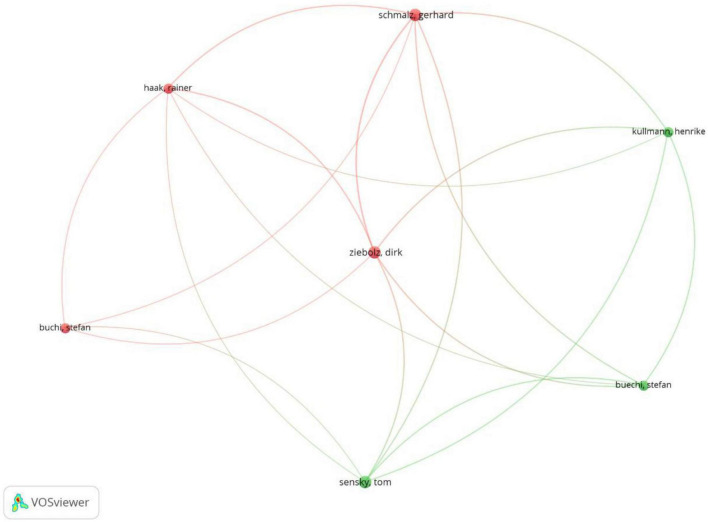
Co-authorship of authors (min number of citations of an author = 2; visualization by weight of documents).

Figure 6 illustrates the partnership network between all the authors as the co-authorship analysis is considered with a minimum of two citations of an author, showing the most influential authors. As is shown, the co-authorship network presents seven prominent authors divided into two clusters. The leading authors in Cluster #1 are Gerhard Schmalz and Dirk Ziebolz, each with three documents and total link strength of 12. In Cluster #2, Tom Sensky is slightly more influential than the other two authors, as he features three documents and total link strength of 10.

**FIGURE 7:**
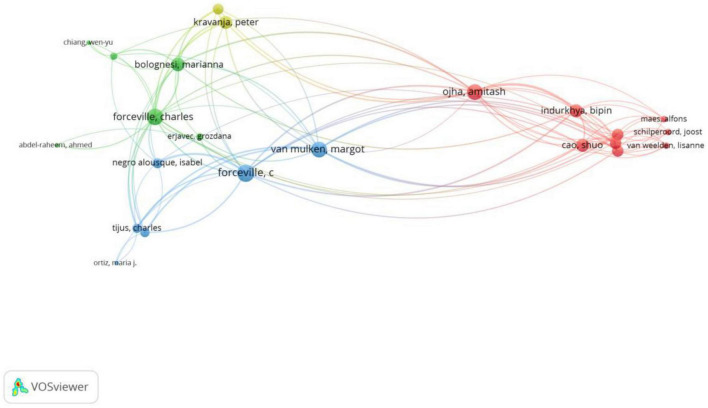
Network of cited authors (min number of citations of an author = 2; visualization by weight of documents).

The citation pattern of the other authors is shown in Figure 7 with a minimum of two citations per author. These authors are grouped into four clusters. Charles Forceville is clearly the most influential author in Figure 7, with the total link strength reaching 76 and the total number of citations standing at 361. It is found that Charles Forceville is the most important author in two clusters generated by VOSviewer, whereas in the remaining two, Amitash Ojha and Peter Kravanja contribute the most with the total link strength of 35 and 22, and the total number of citations, 26 and 11.

#### 3.1.5. Citation: most cited reference, bibliographic coupling

**FIGURE 8:**
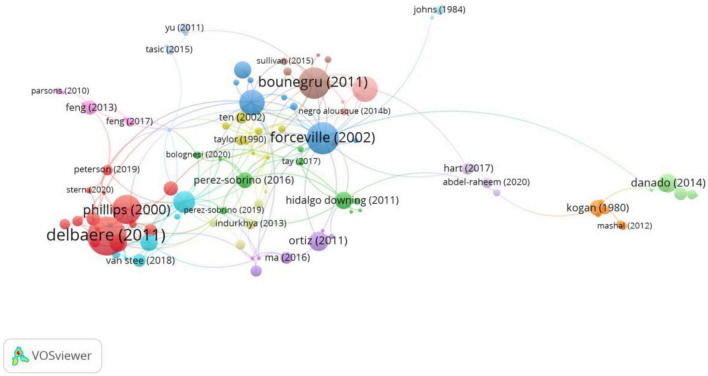
VOSviewer network map showing the most cited reference (min number of citations of a document = 1; visualization by weight of citations).

The citation analysis of documents as revealed in Figure 8 shows there are 15 clusters with a minimum of one citation accounted. As is presented in Table 4, the leading references in each of these clusters are: Kogan et al. (1980), Johns (1984), Forceville (2002), Teng and Sun (2002), Tsakona (2009), van Mulken et al. (2010), Bounegru and Forceville (2011), Delbaere et al. (2011), Hidalgo Downing and Kraljevic Mujic (2011), Ortiz (2011), Yu (2011), Feng and O’Halloran (2013), Indurkhya and Ojha (2013), Danado and Paternò (2014), Hart (2017).

**TABLE 4:** The representative document in the 15 clusters according to citation analysis.

Cluster	References	Article	Journal	Citation
Cluster #1	Delbaere et al., 2011	Personification in Advertising	Journal of Advertising	166
Cluster #8	Bounegru and Forceville, 2011	Metaphors in editorial cartoons representing the global financial crisis	Visual Communication	106
Cluster #10	Tsakona, 2009	Language and image interaction in cartoons: Toward a multimodal theory of humor	Journal of Pragmatics	70
Cluster #5	Ortiz, 2011	Primary metaphors and monomodal visual metaphors	Journal of Pragmatics	41
Cluster #11	Danado and Paternò, 2014	Puzzle: A mobile application development environment using a jigsaw metaphor	Journal of Visual Languages and Computing	39
Cluster #6	van Mulken et al., 2010	The impact of perceived complexity, deviation and comprehension on the appreciation of visual metaphor in advertising across three European countries	Journal of Pragmatics	33
Cluster #2	Hidalgo Downing and Kraljevic Mujic, 2011	Multimodal metonymy and metaphor as complex discourse resources for creativity in ICT advertising discourse	Review of Cognitive Linguistics	32
Cluster #7	Kogan et al., 1980	Understanding Visual Metaphor: Developmental and Individual Differences	Monographs of the Society for Research in Child Development	28
Cluster #9	Feng and O’Halloran, 2013	The visual representation of metaphor	Review of Cognitive Linguistics	23
Cluster #14	Hart, 2017	Metaphor and intertextuality in media framings of the (1984–1985) British Miners’ Strike: A multimodal analysis	Discourse and Communication	17
Cluster #4	Teng and Sun, 2002	Grouping, Simile, and Oxymoron in Pictures: A Design-Based Cognitive Approach	Metaphor and Symbol	16
Cluster #13	Indurkhya and Ojha, 2013	An experimental study on the role of perceptual similarity in visual metaphors	Metaphor Symbol	13
Cluster #11	Yu, 2011	Beijing Olympics and Beijing opera: A multimodal metaphor in a CCTV Olympics commercial	Cognitive Linguistics	13
Cluster #15	Johns, 1984	Visual metaphor: Lost and found	Semiotica	9

**FIGURE 9:**
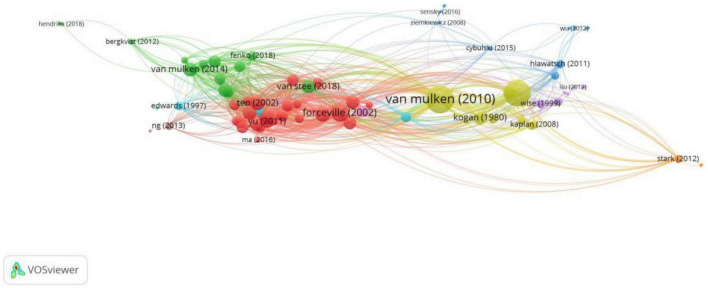
VOSviewer network map showing the bibliographic coupling of documents (min number of citations of a document = 9; visualization by weight of total link strength).

Bibliographic coupling shows the extent to which the documents share the same citations. The network map in Figure 9 shows a total of nine clusters with a minimum of nine citations in one document, as the lowest number of citations among the leading documents listed above is nine. In each of the nine clusters, Forceville (2002) in Cluster #1, van Mulken et al. (2014) in Cluster #2, Hlawatsch et al. (2011) in Cluster #3, van Mulken et al. (2010) in Cluster #4, Wise (1999) in Cluster #5, Tsakona (2009) in Cluster #6, Stark (2011) in Cluster #7, Ng and Koller (2013) in Cluster #8 and Lee (2007) in Cluster #9.

### 3.2. Research domain of co-occurrence: key word plus

**FIGURE 10:**
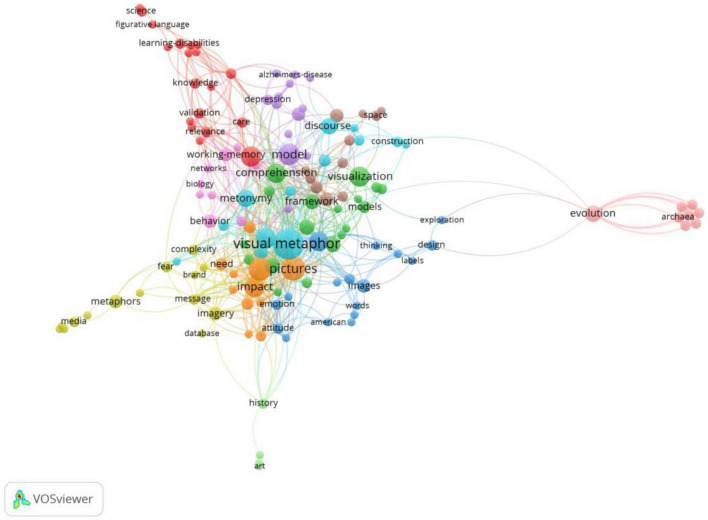
VOSviewer network map showing the co-occurrence of key word plus (mini occurrences of a key word = 2; visualization by weight of occurrences).

The purpose of co-occurrence keyword analysis is to look into the relationship between keywords in a set of publications to uncover the topical issues and help scholars better grasp current research concerns. A total of 549 keywords were investigated, 137 of which appeared more than two times. Figure 10 shows the visual network map of keyword co-occurrence. There are in total 137 items, 11 clusters. The total link strength is 803. The different colored nodes represent different domains of interests in multimodal metaphor. The size of a node implies the occurrence of keywords. The closeness of the relationship between any two items is shown by the thickness of the connection lines.

According to the results of cluster analysis, 11 key areas of research are found: Cluster #1 is related to the visual metaphor comprehension ability of the individuals with intellectual disability. For example, Shnitzer-Meirovich et al. (2018) conduct a program to enhance their analogical thinking and find they can recruit the ability required for visual metaphor comprehension.

Cluster #2 concerns the visualization of the attitudes and the framework for multimodal metaphor comprehension. One of the representative works is Forceville’s (2009) model of multimodal metaphor within a cognitive framework which becomes the mainstream in this field.

Cluster #3 is about the information design and the exploration of the images. For instance, Ojha and Indurkhya (2020) apply perception theory to analyze the design of visual metaphors.

Cluster #4 reveals a perspective from discourse approach to the metaphor representing various emotions. Feng and O’Halloran (2013), integrating social semiotic theory and cognitive linguistic theory, proposes a multimodal discourse approach to explore the structural features of the visual metaphor mapping various emotions.

Cluster #5 concerns the appreciation of visual metaphor in people with old age-related diseases. One of the representative works explores those people’s humor preferences, which finds that they enjoy simple and familiar ones (Kmita et al., 2022).

Cluster #6 is related to the contribution of metonymy to multimodal metaphor or visual metaphor. Those studies demonstrate the unignorable function of metonymy in understanding multimodal metaphor and propose the types of the interaction of multimodal metaphor and metonymy (e.g., Forceville and Urios-Aparisi, 2009).

Cluster #7 focuses on the impact of the persuasion of visual metaphors. Such studies prove that the persuasive effect could be more positive if the message designed in the form of visual metaphor (e.g., Meijers et al., 2019).

Cluster #8 is about space-time metaphor system. In this cluster, the issue is related to use visual metaphor method to understand space-time accessibility. For example, Jiang et al. (2022) use visual metaphor to understand the space-time accessibility of the Hong Kong-Zhuhai-Macao Belt.

Cluster #9 reveals a relation to the contribution of visual metaphor. For example, one of the studies show that the advertisement metaphorically representing the product with personification, appears to lead to more positive brand liking (Marjorie et al., 2011).

Cluster #10 is about the function of visual metaphor in representing the evolution of the eubacteria. Those studies demonstrate the appropriateness of visual metaphor to depict the formation of phenotypic variants of bacterial cells (Sánchez-Romero and Casadesús, 2021).

Cluster #11 concerns the understanding of the art of the images. In this cluster, a representative work is by Poppi et al. (2020), who point out the current interpretations of the metaphorical structures do not always work within the domain of art cognition and thus propose a participant-based framework.

According to the clustering topics, we find that the main issues of multimodal metaphor studies concern what framework to use, what model to interpret, and how to interpret metaphors in different genres, such as advertisements, films, etc. The co-occurrence of the keyword analysis also shows that the effect of multimodal metaphor is one of the hot topics. Those hot topics can be concluded as six top keywords. They are “visual metaphor” with 24 occurrences. “pictures” with 15 occurrences, “persuasion” with 14 occurrences, “model” with 12 occurrences, as well as “impact” and “multimodal metaphor” with 11 occurrences, respectively.

The result implies the current research trend of multimodal metaphor is still in trying to develop an applicable model and in understanding their impacts.

## 4. Discussion

The co-occurrence of the keyword analysis displays that the hot topics related to multimodal metaphor studies could be classified into two research aims. One is related to the framework or the models for the exploration of visual or multimodal metaphor, the other is related to the impact or the power of the multimodal metaphor. The three major approaches driven by the interaction, the conceptual and the blending theories have been used widely and further developed by scholars to study different types of multimodal discourse. The major concern is on the impact of visual metaphor (van Mulken et al., 2010, 33), such as the persuasion of advertisements, cartoons or political discourse (Teng and Sun, 2002; Tsakona, 2009; Hidalgo Downing and Kraljevic Mujic, 2011; Indurkhya and Ojha, 2013). The following sections discuss the research trend and possible future directions in multimodal metaphor studies.

### 4.1. Research direction driven by cognitive linguistic theory

The conceptual multimodal metaphor initiated by Forceville (1996, 2006) has been integrated with social semiotic theory and further developed by Feng and O’Halloran (2013). Feng and O’Halloran (2013) explored the structural features of visual images and models the visual representation of metaphor with respect to the representational, interactive and compositional metafunctions. Their social semiotic model provides a comprehensive account of the visual realization of both creative and conventional metaphors. The model has been widely applied in studies with a social semiotic background and has been used to address the impact of multimodal metaphor in different types of multimodal texts, such as advertisements (e.g., Liu and Zhang, 2020). Nonetheless, more empirical studies concerning more different genres are needed to demonstrate its usefulness. Further, how multimodal metaphor works and how to interpret its power is a research direction in the current research landscape.

### 4.2. Research direction driven by the theory of pragmatics

Within the interaction theory, Ojha and Indurkhya (2016) proposed an improved model for metaphor processing based on the perception theories (O’Regan and Noe, 2001; Zimbardo and Gerrig, 2002) and integrated model of text and image processing (Schnotz, 2002). The model comprises top-down and bottom-up mechanisms, which allow the conceptual and the perceptual features to stimulate each other. Such metaphor features are seen as emergent features. In their model, context is a key role in identifying the source and the target of a visual metaphor. The model has provided implications for the analysis on the design of visual metaphors (e.g., Ojha and Indurkhya, 2020), which would continue to be a direction worth further study.

In the blending theory, Fauconnier and Turner (2002) developed the model into a more systematic, mature and adaptive theory. The three dimensions for the interpretation: composition, completion and elaboration, have been further expanded. The generated emergent structure in the three dimensions is called “running the blend” and is seen as a dynamic and complex cognitive process highly related to the social world. This model thus concerns the pragmatic and sociological interpretation. This model has been applied by Li and Dai (2020) to explore the hidden ideology of print advertisement. It has also been used in the science education field, such as Fredriksson and Pelger (2020). They use it to help students verbalize and visualize abstract phenomena and concepts. Their study demonstrates that the model could support students to understand science matters and their learning process and suggests research directions both in exploiting the use of multimodal metaphors in the education field and in evaluating the use of multimodal metaphors.

### 4.3. Research direction driven by visual/multimodal rhetoric theory

While the above three approaches have illuminated the major directions of multimodal metaphor studies, recent research shows there is another emerging new direction in multimodal metaphor studies. Such studies tend to use the term visual metaphor and draw on rhetoric, pragmatics and argumentation theories, aiming to analyze the impact of multimodal metaphors in, particularly, multimodal arguments. The researchers apply the visual rhetorical theory driven by Barthes’s rhetoric of images, to understand what rhetorical effect of the use of visual metaphor or other rhetorical devices for the reconstruction of argumentation (Kjeldsen, 2018; Tseronis, 2021). The study implies a research tendency of combining multimodal rhetoric theory and argumentation theory to investigate the power of multimodal metaphor in multimodal arguments. The exploration in this field could make clear how multimodal argumentation works as well as how to evaluate them.

In all, the studies within cognitive linguistic theory provide an access to understanding the operation of multimodal metaphor mechanism. The studies within pragmatics accentuate the role of social cultural context and the pragmatic functions of multimodal metaphor. Further, the studies from a visual rhetoric theory are enlightening in integrating conceptual metaphor theory, pragmatics and argumentation theory to explore the rhetorical effect. Those studies also show an interdisciplinary approach to multimodal metaphor research.

To conclude, based on the literature review, we have found that cognitive topics are still the common trend in multimodal metaphor studies. Second, most of the multimodal metaphor studies taking advertisements as data. Further research may include more types of multimodal discourse to understand multimodal metaphor more systematically and therefore to further improve current modes for multimodal metaphor interpretations. Third, the impact of multimodal metaphors in multimodal arguments is a new field worth further exploration. Last, more works should be done using multimodal corpus analysis and empirical approaches to prove the applicability of various models.

## 5. Conclusion

Multimodal metaphor including metaphor constructed in non-verbal expressions, i.e., pictorial/visual metaphor is an important concept as it is based on theoretical mechanisms of interaction, blended space and conceptual blending and is applicable in many practical scenarios such as advertisement and animation. While multimodal metaphor has been a prospering concept that has been increasingly investigated, there remain few studies that address its research trend through a bibliometric analysis.

Based on the 397 articles obtained from the Web of Science (WoS) Core Collection Database in all relevant editions, the current study reveals significant patterns in publication features including publication years, country/region participation, journal publication, participating authors, citation and research domain of concurrence. It is found that there has been a general rising trend in the research on multimodal metaphor with 2020 the most fruitful year. USA is the country with the highest number of documents and collaboration links. Journal of Advertising and Journal of Pragmatics are two most influential sources in terms of the number of citations and co-citations. With minimal co-authorship with others, Charles Forceville is the most impactful author as far as the total link strength and the total number of citations are concerned. This has been confirmed by citation analysis of most cited reference and bibliographic coupling. Co-occurrence in key word plus suggests 11 research domains related to what framework to use, what model to interpret, and how to interpret metaphors in different genres.

In addition to the two research aims concluded from the most frequent hot topics, we have discussed three research directions driven by three different strains of theories, while they are all cognitive in nature. Our findings suggest that the research trends in existing research on multimodal metaphor lie in the types of multimodal discourses, the investigation in multimodal arguments and the use of multimodal corpus analysis.

This study can be potentially useful for those attempting to contribute to the existing line of research, as it provides a detailed account of the entire landscape of literature on multimodal metaphor and sheds light on the possible research directions for further exploration. However, there are limitations that should be noted. First, only one source of data is considered. Future research can involve other sources of data such as Google Scholar and Scopus to generate a more extending view. Second, we have only used VOS viewer as the only bibliometric tool, which means the aspects discussed on the scholarship collected are restricted. It is possible to employ other similar tools such as CiteSpace and Network Workbench to look into more features of the publications.

## Data availability statement

The original contributions presented in this study are included in the article/supplementary material, further inquiries can be directed to the corresponding author.

## Author contributions

ZZ compiled the data and did the statistics and was devoted to chapters 2, 3.1 and conclusion. SW was devoted to chapters 1, 3.2, and 4. SC participated in the discussion and proofread the whole manuscript. All authors annotated the data, contributed to the article, and approved the submitted version.
